# The physiology of drought stress in grapevine: towards an integrative definition of drought tolerance

**DOI:** 10.1093/jxb/eraa245

**Published:** 2020-05-20

**Authors:** Gregory A Gambetta, Jose Carlos Herrera, Silvina Dayer, Quishuo Feng, Uri Hochberg, Simone D Castellarin

**Affiliations:** 1 EGFV, Bordeaux-Sciences Agro, INRA, Université de Bordeaux, ISVV, chemin de Leysotte, Villenave d’Ornon, France; 2 Institute of Viticulture and Pomology, Department of Crop Sciences, University of Natural Resources and Life Sciences Vienna (BOKU), Tulln, Austria; 3 Wine Research Centre, Faculty of Land and Food Systems, The University of British Columbia, Vancouver, BC, Canada; 4 ARO Volcani Center, Institute of Soil, Water and Environmental Sciences, Rishon Lezion, Israel; 5 Hong Kong Baptist University

**Keywords:** Agriculture, climate change, fruit ripening, viticulture, water deficit, wine

## Abstract

Water availability is arguably the most important environmental factor limiting crop growth and productivity. Erratic precipitation patterns and increased temperatures resulting from climate change will likely make drought events more frequent in many regions, increasing the demand on freshwater resources and creating major challenges for agriculture. Addressing these challenges through increased irrigation is not always a sustainable solution so there is a growing need to identify and/or breed drought-tolerant crop varieties in order to maintain sustainability in the context of climate change. Grapevine (*Vitis vinifera*), a major fruit crop of economic importance, has emerged as a model perennial fruit crop for the study of drought tolerance. This review synthesizes the most recent results on grapevine drought responses, the impact of water deficit on fruit yield and composition, and the identification of drought-tolerant varieties. Given the existing gaps in our knowledge of the mechanisms underlying grapevine drought responses, we aim to answer the following question: how can we move towards a more integrative definition of grapevine drought tolerance?

## Teaser

Review of grapevine drought responses and the search for drought-tolerant varieties that can increase the sustainability of viticulture in the context of climate change.

## Introduction

Grapevine (*Vitis vinifera*) is one of the most economically important fruit crops worldwide (Alston and Sambucci, 2019), and its use in wine production has played an important cultural role in many parts of the world. Grapevines are now cultivated in more than 90 countries for wine, distilled liquors, juice, table grapes, and raisin production (FAO-OIV, 2016). Because of its global economic importance, the climatic diversity of the producing regions, and the large number of studies (from genomics to production practices), grapevine has emerged as a model perennial fruit crop species.

Almost all wine regions in the world are located in temperate zones and many have a Mediterranean climate characterized by warm and dry summers. In these regions grapevines are regularly exposed to periods of drought unless irrigation is applied, and currently much of the world’s winegrape production is not irrigated. For instance, as of 2016, fewer than 10% of vineyards in Europe were irrigated (Costa *et al.*, 2016). However, this percentage appears to be increasing for a variety of reasons including climate change and the relaxation of irrigation restrictions in many traditionally rainfed regions. For example, in Spain the irrigated vineyard area had increased from ~2% in the 1950s to almost 27% in 2015 (Ayuda *et al.*, 2020), and looking at the most recent data from Spain in 2018, that percentage has grown further to ~30% (data obtained from the ‘Anuario de Estadística’, Spanish Ministry of Agriculture). Water deficits impair vine growth and decrease yield, but can improve grape and wine quality unless they are severe (Chaves *et al.*, 2010). In regions where irrigation is used, much attention is placed on developing deficit irrigation strategies (i.e. application of irrigation at levels below what would be required to sustain 100% evapotranspiration) with the goal of producing high quality grapes, particularly for red wines, while minimizing yield losses. The relative importance of yield versus quality varies with the type of grapes being produced and the producer’s goals.

Studies indicate that climate change will exacerbate drought events in many traditional wine growing regions, which will likely increase the need for irrigation (Intergovernmental Panel on Climate Change, 2014). Many argue that irrigation is not a sustainable solution, and although this is likely true for water-scarce regions, putting concrete numbers on this assertion is extremely difficult (Hannah *et al.*, 2013). Quantitative studies that predict the increase in freshwater resource consumption by expanding vineyard irrigation are needed in many regions.

Because of the lingering questions surrounding the sustainability of irrigation, there has been a strong focus on the need to understand the differences in drought tolerance between existing grapevine varieties (Medrano *et al.*, 2018). The logic is that a detailed mechanistic understanding of the differences in drought tolerance between existing genotypes would (i) increase sustainability through informing the optimum choice of plant material for a given climate-production context and (ii) identify the key traits responsible for these differences in order to better design and target phenotyping approaches for the development of new drought-tolerant varieties. It is important to keep in mind that grapevines are perennial crops that typically produce for many decades. Thus, drought tolerance from the point of view of viticulture must comprise both the ability to maintain productivity within the current season and equally the ability to avoid negative carry-over effects, and perhaps drought-induced mortality, across many seasons (Gambetta, 2016).

In order to characterize the differences in drought tolerance between grapevine genotypes, many studies have focused on key agronomic indicators such as yield and grape composition (i.e. fruit quality), while others have focused on finer scale physiology such as stomatal regulation and carbon assimilation. Despite extensive study, how different varieties respond to drought in terms of water use, growth, fruit quality, and yield remains an open and critical question for managing water in vineyards. Yield is impaired under drought conditions but a recent meta-analysis suggests this decrease may be variety specific (discussed below; Mirás-Avalos and Intrigliolo, 2017). Recent work suggests that all genotypes regulate vine water use (i.e. stomatal conductance) in order to protect against more severe damage such as petiole or leaf cavitation and leaf shedding (Hochberg *et al.*, 2017*c*; Dayer *et al.*, 2020). However, it is unclear to what extent differences in the regulation of vine water use between varieties result from innate genotypic differences, as was traditionally thought, or environmental factors (Hochberg *et al.*, 2018). Furthermore, grapevine appears to almost always operate within a ‘safe’ margin of water potentials in which stem cavitation is extremely rare (Charrier *et al.*, 2018), but we still do not know the exact mortality thresholds for grape. Thus, numerous gaps remain in our understanding of what really constitutes a drought-adapted grapevine variety, making it difficult to robustly address future climate challenges. Given the existing gaps in our knowledge, this review focuses on the following question: How can we move towards a more integrative definition of grapevine drought tolerance? Accordingly, we synthesize the current state-of-the-art regarding grapevine drought stress physiology, including the impact on leaves and fruit composition, and use this background to discuss an integrative approach to define and characterize drought-adapted varieties.

### Grapevine drought responses

#### Stomatal regulation of vine water use

**Fig. 1.**
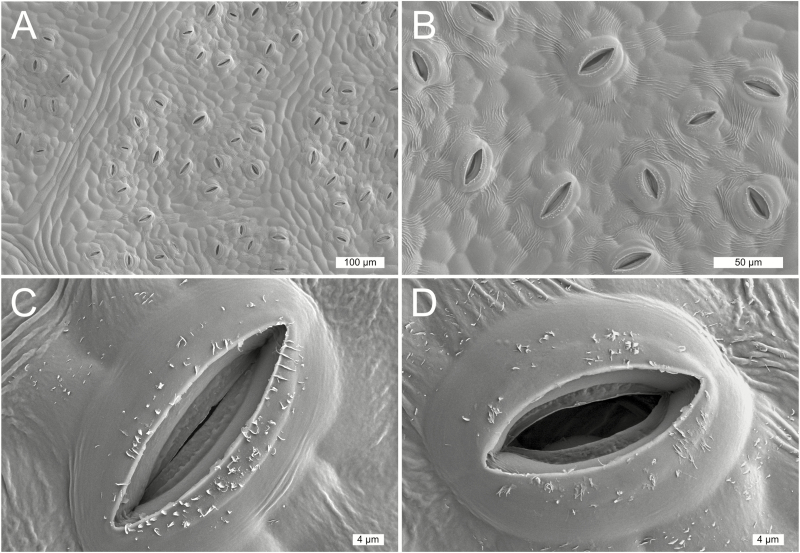
Cryo-scanning electron microscopy images of the underside of a grapevine (*Vitis rupestris*) leaf at various magnifications. Islands of stomata (A) are visible in between leaf vascular traces, and there is ample aperture diversity (B) of stomata in more closed (C) or open (D) states. Scale bars are shown in each panel.

According to the cohesion–tension theory (reviewed by Tyree and Zimmermann, 2002), water flows from the soil to the atmosphere through the plant xylem network under tension (i.e. under a negative pressure), pulled by the more negative water potentials (Ψ) in the leaf tissues where water is transpired into the atmosphere through stomata (Fig. 1). By regulating its stomatal and hydraulic conductance the plant can determine its Ψ anywhere between the soil water potential and the atmospheric water potential. In grapevines, Ψ usually ranges between −0.3 and −2.0 MPa (e.g. Charrier *et al.*, 2018). Because the water flows in a metastable state (i.e. under tension), when the tension exceeds certain limits, cavitation occurs, leading to hydraulic failure and the risk of mortality (Tyree and Sperry, 1989).

**Fig. 2.**
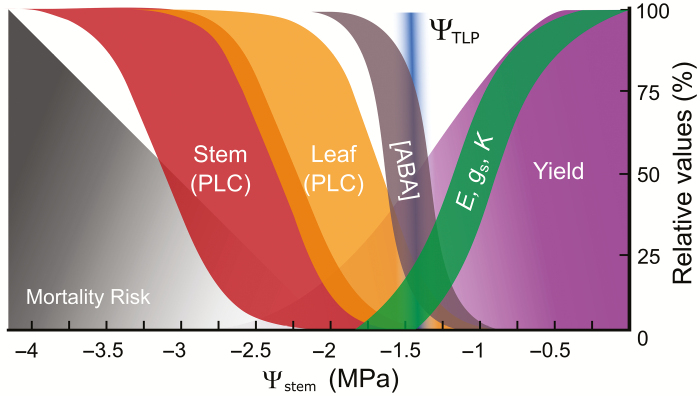
The sequence and thresholds of grapevine responses to water deficit. Irrigated vineyards typically function within a safe range of water potentials (green transpiration zone, Ψ _stem_>−1.5 MPa). In this zone grapevines can experience decreased transpiration (*E*), stomatal conductance (*g*_s_), hydraulic conductance (*K*), photosynthesis, and fruit yield (purple). ABA concentration ([ABA]) does not increase until after stomata are mostly closed. As water deficit increases vines can acclimate through numerous processes. One is osmotic adjustment where their leaf turgor loss point (Ψ _TLP_, blue) can become more negative allowing the vine to operate across a wider range of Ψ _stem_. If Ψ _stem_ becomes increasingly negative (~Ψ _stem_<−1.6 MPa) grapevines can experience cavitation producing embolisms in the water-conducting xylem vessels (quantified as the percentage loss of conductivity, PLC). Grapevines exhibit ‘vulnerability segmentation’ in which leaves (orange) are more vulnerable to embolism than perennial tissues (e.g. trunks and stems; red). Embolism in leaves acts as a hydraulic fuse protecting perennial organs from experiencing levels of water stress that would lead to embolism. Extensive embolism can lead to vine mortality (grey).

Stomata are key players in a plant’s response to drought. During drought stomata close to reduce transpiration, avoid critical Ψ, and conserve water (Darwin, 1898). Without this reduction in transpiration, the fast flow could lead to large pressure drops between the soil and leaf (i.e. increasingly negative Ψ). Irrigated vineyards typically function within a safe range of water potentials (Fig. 2, Ψ _stem_>−1.5 MPa) that do not lead to cavitation or turgor loss. Even non-irrigated vineyards seldom surpass these values (Charrier *et al.*, 2018). When vineyard managers utilize deficit irrigation strategies, most commonly in association with premium red winegrape production, they normally target levels of water deficit (Ψ _stem_ −1.2 to −1.4 MPa) that are certainly great enough to decrease stomatal conductance (*g*_s_), transpiration, photosynthesis, and ultimately fruit yield (Fig. 2). More severe water deficit (Ψ _stem_<−1.6 MPa) might induce turgor loss and xylem cavitation that could lead to leaf shedding and even vine mortality.

Different grapevine genotypes exhibit a continuum of stomatal sensitivities to water deficit (Lavoie-Lamoureux *et al.*, 2017; Charrier *et al.*, 2018; Levin *et al.*, 2019; Dayer *et al*., 2020) where some varieties tend to close their stomata earlier than others (discussed below). Regardless of these differences all vines close their stomata in response to water deficit within a relatively narrow range of water potentials when compared with the breadth of plant taxa in general (e.g. compare Lavoie-Lamoureux *et al.*, 2017; Martin-StPaul *et al.*, 2017). Understanding the underlying mechanisms that drive the regulation of stomata under drought allows the identification of targets that could be leveraged in the breeding of more drought-tolerant varieties and/or rootstocks.

#### The mechanisms contributing to stomatal regulation

One of the reasons why the elucidation of the mechanisms driving stomatal regulation remains so challenging is that they are numerous and interdependent, involving biochemical and hydraulic signals among others (Buckley, 2019). The most prominent biochemical player studied is the plant hormone abscisic acid (ABA), which has become synonymous with drought stress, although ABA functions in numerous plant processes. ABA directly affects *g*_s_ at the guard cell level, and the molecular mechanisms driving these direct effects are well studied. ABA binds to the PYR/PYL/RCAR ABA receptors that interact with, and remove the inhibitory action of, the 2C protein phosphatases (PP2Cs) (Nakashima and Yamaguchi-Shinozaki, 2013). Other molecular messengers eventually activate ion efflux transporters that decrease guard cell turgor and close the stomata (Boneh *et al.*, 2012; Munemasa *et al.*, 2015). Changes in Ψ _leaf_ and/or hydraulic conductance (*K*) also affect guard cell turgor and *g*_s_. Changes in *K* likely involve the regulation of aquaporins, cell membrane proteins that facilitate water and small molecule transport through plasma membranes (e.g. Gambetta *et al.*, 2017; Maurel and Prado, 2017). Aquaporins comprise a large gene family whose regulation is complex, and they are expressed in all grapevine tissues (Wong *et al.*, 2018). As in other plant species, aquaporins have been linked to grapevine stomatal regulation and to differences in this regulation between varieties (Vandeleur *et al.*, 2009; Vitali *et al.*, 2016; Coupel-Ledru *et al.*, 2017).

The relative contribution of these biochemical and hydraulic mechanisms is still unknown and likely dependent on genotype (McAdam and Brodribb, 2015; Coupel-Ledru *et al.*, 2017; Peccoux *et al.*, 2018) as well as environment (Lovisolo *et al.*, 2016; Hochberg *et al.*, 2017b). When the entire breadth of plant taxa is considered, we observe a spectrum of relative contributions of these two modes (hydraulic and biochemical) from gymnosperms that utilize a purely hydraulic mode to angiosperms whose regulation is largely ABA-dependent (McAdam and Brodribb, 2015). In grapevine, studies suggest that both hydraulics and ABA play a role in stomatal regulation although the relative contribution of these two modes remains unclear (Tombesi *et al.*, 2015; Peccoux *et al.*, 2018). Studies in grape have found that ABA increases only when *g*_s_ falls to very low levels (≤50 mmol m^−2^ s^−1^), suggesting that early stomatal closure is not ABA driven (Fig. 2 ; Pou *et al.*, 2008; Hochberg *et al.*, 2013b; Tombesi *et al.*, 2015; Degu *et al.*, 2019).

The hydraulic and biochemical modes of stomatal regulation are interdependent, making a strict division between the two extremely challenging both theoretically and experimentally. A good example of this conundrum is illustrated by the links between ABA and *K*. Changes in Ψ are modulated in part through changes in *K* and, in turn, *K* is modulated by ABA, creating an interdependence that has yet to be disentangled. This interdependence appears to be present in most plant organs. For example, leaf hydraulic conductance (*K*_leaf_) is modulated by ABA (Shatil-Cohen *et al.*, 2011; Pantin *et al.*, 2013). In grape specifically, genotypes appear to differ in the sensitivity of *K*_leaf_ to exogenous ABA treatments (Coupel-Ledru *et al.*, 2017). This implies that when ABA concentrations increase in leaves under water deficit there are effects on both *g*_s_ and *K*, both of which when integrated would affect Ψ _leaf_, and thus ABA, setting up a circular loop. Furthermore, cell shrinkage was shown to increase ABA biosynthesis (McAdam and Brodribb, 2016), suggesting the reduction in Ψ _leaf_ would reinforce the ABA effect on the stomata. The same situation is present in roots, where root hydraulic conductance also appears to be modulated by ABA (reviewed in Gambetta *et al.*, 2017). The challenge of this complexity is not restricted to the examples highlighted above and researchers have suggested a number of other mechanisms including changes in guard cell ABA sensitivity as a function of Ψ _leaf_ (distinct from changes in ABA concentration) and other potential biochemical signals like pH (Chaves *et al.*, 2010; Geilfus, 2017) and reactive oxygen species (Cruz de Carvalho, 2008; Miller *et al.*, 2010).

#### Decreases in photosynthesis

During drought, photosynthesis is limited by both stomatal closure and impairment of the photosynthetic machinery (i.e. metabolic factors). The relative contributions of these two mechanism are not always clear and an in-depth discussion of these factors is outside the scope of the current review (see the review by Flexas *et al.*, 2004). In grape, the photosynthetic machinery appears to be very tolerant to mild, and even medium, levels of water deficit (Flexas *et al.*, 1998; Chaves *et al.*, 2010; Dayer *et al.*, 2019). For example, Flexas *et al.* (2002) demonstrated that *g*_s_ needed to be reduced by 60–70% before changes in electron transport rate and non-photochemical quenching of chlorophyll fluorescence were observed. Intrinsic water use efficiency (WUEi) increases under mild drought, and then later decreases under more severe or long-term drought because of the damage or inhibition of photosynthesis (Bota *et al.*, 2016). It seems that non-stomatal limitations become dominant only when grapevine *g*_s_ falls below 50 mmol m^−2^ s^−1^ (Flexas and Medrano, 2002).

Under long-term drought, mesophyll conductance decreases, negatively affecting CO_2_ diffusion and limiting photosynthesis (Ouyang *et al.*, 2017). In grape, decreases in mesophyll conductance appear to be coincident with an impairment of the photosynthetic machinery (Flexas *et al.*, 2002), although the exact magnitude and timing of the decrease is likely variety specific and related to leaf anatomical structure (Tomás *et al.*, 2014). Research in other species has demonstrated that modulation of mesophyll conductance results in part from the regulation of leaf aquaporin expression and/or activity (Maurel and Prado, 2017) and the same is also likely true in grape (Flexas *et al.*, 2010).

Drought exacerbates photoinhibition (i.e. a transient decrease of the photochemical efficiency of PSII as a result of excess photosynthetic photon flux density), and different varieties have developed various strategies to cope with this (Guan *et al.*, 2004). Beis and Patakas (2012) in a study of Greek *Vitis vinifera* varieties found that the drought-tolerant Sabatiano removed excess absorbed light by thermal dissipation (non-photochemical quenching) and protected cells via rapid activation of the antioxidant defense system. In contrast, Mavrodafni relied on the activation of photorespiration, which is an alternative process to dissipate excess light energy and thus prevent damage to the photosynthetic apparatus. Hochberg *et al.* (2013a) observed a link between photosynthetic efficiency and photorespiration rate and stomatal regulation when comparing Cabernet Sauvignon and Syrah. Cabernet Sauvignon displayed tighter stomatal control and higher photosynthesis and photorespiration rate, which may help ameliorate the photoinhibition caused by the stomatal closure.

#### Leaf osmotic adjustment

The concentration of solutes in the symplast enables leaves to maintain turgor pressure under negative Ψ (Hsiao *et al.*, 1976). For this reason, the Ψ at which leaf cells lose turgor (i.e. turgor loss point; Ψ _TLP_) has been used as a leaf drought tolerance trait across species. Leaf turgor loss is correlated with stomatal closure (Brodribb *et al.*, 2003) and the decline in *K*_leaf_ (Scoffoni *et al.*, 2011; Trifiló *et al.*, 2016) so that leaves with a more negative Ψ _TLP_ are able to maintain stomatal and hydraulic conductance and growth under drier conditions (Bartlett *et al.*, 2012). The same relationships between Ψ _TLP_ and stomatal closure have been found in grapevines (Fig. 2; Hochberg *et al.*, 2017*a*; Dayer *et al.*, 2020).The Ψ _TLP_ is a plastic trait and most plant species decrease the osmotic potential under drought conditions by accumulating osmotically active solutes in leaf cells (i.e. osmotic adjustment). This allows them to maintain positive cell turgor despite decreasing Ψ (Bartlett *et al.*, 2014; Maréchaux *et al.*, 2017).

Previous research has shown that grapevines osmotically adjust in response to drought conditions (Downton and Millhouse, 1983; During, 1984; Rodrigues *et al.*, 1993; Schultz and Matthews, 1993; Patakas and Noitsakis, 1999; Patakas *et al.*, 2002; Martorell *et al.*, 2015; Hochberg *et al.*, 2017*a*). However, it is important to mention that different species accumulate different osmolytes (Sofo *et al.*, 2008; Warren *et al.*, 2012) and little information is available regarding the compounds involved in the osmoregulation of grapes. Nitrogen metabolism, particularly amino acids, is responsive to drought stress in grapevine and might have a role in the osmotic adjustment (Hochberg *et al.*, 2013b). Proline has been suggested as a prominent osmolyte because of its accumulation under stress, but it seems that its contribution to the osmotic potential is marginal. Patakas *et al.* (2002) and more recently Degu *et al.* (2019) suggested that calcium accumulation could have a pivotal role in the osmotic adjustment of grape leaves together with other inorganic ions such as potassium. Interestingly, some authors found an increase in calcium oxalate crystals in leaves of grapevines under drought, suggesting that these structures in the mesophyll could either play a functional role in water stress and calcium regulation, or represent an unintended result of the increased calcium accumulation (Liu *et al.*, 2015; Doupis *et al.*, 2016).

#### Hydraulic vulnerability segmentation

**Fig. 3.**
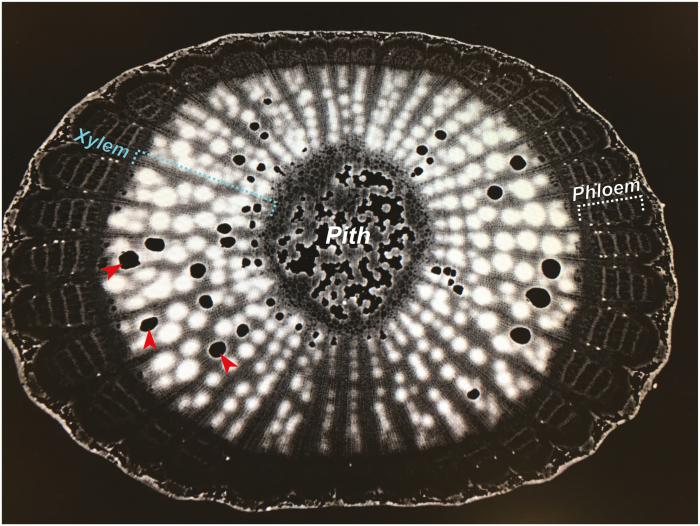
Non-invasive visualization of embolisms in a 1-year-old grapevine cane using high-resolution computed tomography. Functional xylem vessels appear bright white (visualized via the contrasting agent iohexol), but some vessels have become embolized (black xylem vessels, examples shown with red arrowheads) and do not conduct water.

Severe water deficits can increase the tension in the water column to levels at which embolisms form within the xylem vessel rendering the xylem conduit in which they occur permanently or temporarily dysfunctional (Fig. 3). Widespread embolism (hydraulic failure) can lead to leaf shedding and vine mortality. Grapevines, however, exhibit a special trait referred to as hydraulic vulnerability segmentation, which protects the perennial tissues (e.g. trunks and canes) from experiencing levels of water stress that would lead to embolism (Tyree and Ewers, 1991). Plants exhibit hydraulic vulnerability segmentation when distal organs, such as leaves, are more vulnerable to embolism than the perennial organs. As Ψ becomes more and more negative, petioles and leaves experience embolism first, severing the water column connecting leaves to the perennial organs. This acts as a ‘hydraulic fuse’ protecting the perennial organs from more negative Ψ (Tyree and Ewers, 1991). Many species exhibit vulnerability segmentation (Tsuda and Tyree, 2000; Pivovaroff *et al.*, 2014; Peguero-Pina *et al.*, 2015; Johnson *et al.*, 2016), conferring the ability to conserve their vital organs in the face of severe drought.

In grapevine, studies using destructive methods to measure hydraulic conductivity have suggested the presence of vulnerability segmentation (Schultz, 2003; Lovisolo *et al.*, 2008; Choat *et al.*, 2010; Tombesi *et al.*, 2014; Shelden *et al.*, 2017). Recent studies using non-destructive *in vivo* visualization via magnetic resonance imaging and synchrotron-based microcomputed tomography revealed the same vulnerability segmentation (Hochberg et al., 2016*a*, 2017*c*; Charrier *et al.*, 2016). When synthesized, studies in grapevine demonstrate that petioles and leaves are significantly more vulnerable than stems (Fig. 2). Further, the hydraulic vulnerability of basal leaves is greater than that of the apical leaves, which enables grapevines to preferentially shed the older and less photosynthetically efficient basal leaves (Hochberg *et al.*, 2017*c*). The vulnerability segmentation strategy suggests that under severe drought grapevines have a ‘wait it out’ strategy, in which the vine is designed to abandon the current year’s canopy to preserve the perennial structure until the following season.

The mechanisms that bring about vulnerability segmentation in grape are not understood. Generally, larger xylem vessels are more susceptible to embolism than smaller vessels due to higher inter-vessel pit surface areas and more pits (Wheeler *et al.*, 2005). However, the xylem vessel diameter of the petioles was smaller than that of stems (Hochberg *et al.*, 2016*a*), which implies that petioles would be more resistant to embolism than the stem solely based on this criterion. Hochberg *et al.* (2016*a*) proposed that the larger proportion of primary xylem than secondary xylem in petioles may, in part, explain this discrepancy as primary xylem is more embolism sensitive. Differences in pit pore properties, xylem vessel arrangement, along with other factors may also contribute to the segmentation (Holbrook and Zwieniecki, 2005). More research is needed to identify the source of the observed vulnerability differences.

#### Extreme drought

High levels of embolism in perennial organs, referred to as ‘hydraulic failure’, can lead to plant mortality (McDowell *et al.*, 2008). Although their vulnerability segmentation protects against high levels of embolism in stems and trunks, grapevines can still die from drought. Extreme water deficits (Fig. 2, Ψ _stem_<−2 MPa) generally result in the loss of much or all of the canopy and crop in the current season. The following season the vine may have the potential to regrow if some buds have survived the drought episode although the exact desiccation thresholds of bud tissues are unknown. In potted-plant experiments even when vines are stressed to levels that result in nearly complete defoliation and 100% loss of conductivity due to embolism in stems, a large percentage of vines still regrow the following season (Tombesi *et al.*, 2018; G. Charrier and G. A. Gambetta, unpublished data). The ability of vines to recover from and/or repair extensive embolism over winter may involve their ability to refill embolized xylem vessels in the stem (Knipfer *et al.*, 2015a; Nardini *et al.*, 2017). However, leaves and petioles do not appear to recover from embolism. Embolized petioles were not refilled even when submerged overnight in water (Hochberg *et al.*, 2016b) and embolized leaves were shed even after irrigation was administered and stress was relieved (Hochberg *et al.*, 2017*c*).

Assuming a vine does recover over winter from an extreme drought event there are still many questions regarding the extent to which the vine may be compromised for future events and how future fruit yields could be impacted in subsequent seasons. One concern is cavitation fatigue, in which previous cavitation events lead to an increase in vulnerability to cavitation (Hochberg *et al.*, 2017*a*). It is reasonable to assume that vines that have undergone a drought event that resulted in severe levels of embolism may be more vulnerable to future events. With respect to season-to-season carry-over effects on fruit yield, even less extreme water deficits can sometimes lead to decreased yields in the following season (Buttrose, 1974; Williams and Matthews, 1990; Dayer *et al.*, 2013), but to date few studies have investigated the carry-over effects of more severe drought events (Tombesi *et al.*, 2018).

### Defining drought tolerance in grapevine

The hydraulic variability between grapevine cultivars has been well known for several decades and has been the subject of dozens of studies (e.g. Schultz, 2003; Rogiers *et al.*, 2012). Different cultivars have been shown to have different sensitivities to deficit irrigation in terms of changes in fruit yield and composition (e.g. Hochberg *et al.*, 2015a; Martorell *et al.*, 2015). It is critical to understand the physiological source of this variability in order to improve predictions of varietal behavior in response to climate and to optimize irrigation strategies. Unfortunately, there are numerous hydraulic traits that determine a vine’s response to water availability: xylem architecture (Hochberg *et al.*, 2015*b*), aquaporin regulation (Vandeleur *et al.*, 2009), ABA dynamics (Soar *et al.*, 2006), osmotic adjustment (Martorell *et al.*, 2015), root characteristics (Alsina *et al.*, 2011), and stomatal regulation (Schultz, 2003) are all linked to hydraulic variability between cultivars. Even if we were to explore all of these traits in a single cultivar (which to date has never been done), transformation of the resulting physiological traits matrix into a hydraulic strategy exceeds our current physiological models. Therefore, to simplify cultivars’ hydraulic classification, researchers turned to a phenomenological scale (Tardieu and Simonneau, 1998) ranking species from isohydric to anisohydric.

The iso/anisohydric terminology was introduced into grapevine hydraulics by Schultz (2003), who used this terminology to distinguish between Grenache and Syrah based on seasonal water potential (Ψ) homeostasis. Later, the terms gained additional meanings, describing daily Ψ homeostasis (Soar *et al.*, 2006) or the Ψ leading to stomatal closure (Lavoie-Lamoureux *et al.*, 2017). The classification became popular since it was relatively easy to measure based on a few physiological traits (i.e. *g*_s_ and Ψ) and could presumably indicate the variety’s performance under drought or the underlying physiological traits (e.g. hydraulic vulnerability (Schultz, 2003), aquaporin expression (Vandeleur *et al.*, 2009), and transciptomic responses (Dal Santo *et al.*, 2016). However, it can be argued that the iso/anisohydric paradigm has not provided much information about cultivars’ response to water stress. A case in point, after 17 years, with over 80 grapevine studies that have used the terminology, it is still unclear which of the two (iso/anisohydric) represents cultivars that are better adapted to drought.

Recently, Hochberg *et al.* (2018) pointed out that the use of the iso/anisohydric terminology should be abandoned for two reasons: (i) the different definitions (seasonal Ψ homeostasis, daily Ψ homeostasis, and stomatal regulation) are not necessarily in agreement with one another, creating confusion as to the actual meaning of the terms; and (ii) the environmental effects are at least as significant as the genotypic effect, and thus we cannot predict a cultivar’s hydraulic behavior without accounting for the environment (Hochberg *et al.*, 2018;  Villalobos-González *et al.*, 2019). It is important to explicitly state that the call to abandon the terminology is not tantamount to a denial of the hydraulic variability between grape cultivars. It only stresses that we should seek other approaches in order to more accurately describe, and better predict, varietal responses to water deficit.

The environmental effect on varietal responses could be approached through large cultivar comparisons in common garden experiments (e.g. Gowdy *et al.*, 2018) or phenotyping facilities (e.g. Coupel-Ledru *et al.*, 2014; Charrier *et al.*, 2018; Dayer *et al.* 2020). In addition to the effects that directly originate from environmental factors (soil water potential, soil hydraulic conductivity, and vapor pressure deficit), the exposure to different environments will also shape the vine’s hydraulic properties (i.e. hydraulic plasticity; Lovisolo *et al.*, 2010). Turgor loss point, stomatal regulation, and hydraulic architecture were all shown to have high environmental plasticity (Lovisolo *et al.*, 2010; Martorell *et al.*, 2015; Hochberg et al., 2015*b*, 2017*a*; Munitz *et al.*, 2018), meaning that cultivar comparisons under different conditions, and at different developmental stages, might not always lead to the same hydraulic behavior. This may be one reason for the variable behaviors that have been observed in the same variety between different studies (e.g. Chaves *et al.*, 2010; Charrier *et al.*, 2018). Common garden experiments or phenotyping facilities should reveal the hydraulic differences that originate from genotypic variability and provide the hydraulic base that could be later expanded into plasticity studies.

To move beyond the iso/anisohydric terminology we propose to describe a specific variety’s drought tolerance with the following four core physiological traits that when integrated describe a vine’s response to water stress.

(i) Maximal transpiration rate (*E*_max_), a good indicator for the soil reservoir depletion rate. A rapidly transpiring vine is more likely to experience stress in between water supplies (either rain or irrigation). Measurements should be made on healthy well-watered vines and always referenced to the potential evapotranspiration (Penman, 1948). *E*_max_ could be estimated indirectly through a combination of leaf area and *E* measurements for specific leaves, but those are likely to overestimate the actual *E*_max_ due to the large variability in *E* between leaves (Escalona *et al.*, 2016). Alternatively, *E*_max_ could be evaluated from lysimeter measurements. In field scale lysimeters *E*_max_ ranged between 4 and 60 litres day^−1^, depending on leaf area and potential evapotranspiration (Williams and Ayars, 2005). However, due to the heavy cost of field scale lysimeters, large cultivar comparisons of *E*_max_ are more feasible in small pots (Coupel-Ledru *et al.*, 2014).
(ii) Stomatal regulation, one of the most widely studied traits impacting drought tolerance. It is expressed as the *g*_s_–Ψ _leaf_ curve and indicates the maximal photosynthetic capacity (Medrano *et al.*, 2003; Brodribb *et al.*, 2020) as well as the critical Ψ _leaf_ that plants are trying to avoid (Meinzer *et al.*, 2009; Choat *et al.*, 2018). The latter could be used to calculate the soil water reservoir that is available to the vine (in combination with root volume and the soil water retention curve). Of the four core traits, stomatal regulation is by far the most widely studied, probably due to the relative simplicity of the Ψ _leaf_ and *g*_s_ measurements. However, curve fitting and its extrapolation to predict Ψ _crit_ are not trivial, especially when attempting to determine the Ψ _leaf_ that leads to stomatal closure. Since the curve tends to *g* _s_=0, the error when calculating the Ψ _leaf_ that leads to 1% *g*_s_ (compared with the maximal *g*_s_) might be in the magnitude of several MPa. Therefore, it is recommended to select values from the center of the curve (Ψ _leaf_ that leads to 10–25% of the maximal *g*_s_) that could serve as proxies for stomatal closure (Lavoie-Lamoureux *et al.*, 2017). In a meta-analysis of 40 studies, Lavoie-Lamoureux *et al.* (2017) showed that at −1.2 MPa, *g*_s_ varied between 45 and 338 mmol m^−2^ s^−1^, reflecting the large variability of this trait. Interestingly, these authors found that studies that used porometers measured on average two times higher *g*_s_ compared with studies that used infra-red gas analysers (Lavoie-Lamoureux *et al.*, 2017). It is clear that measuring the actual *g*_s_ is not as trivial as portrayed by commercial producers of gas exchange devices, and that in addition to using best practices such as making comparisons using the same instrument within studies, substantial variation should be expected between studies.
(iii) Turgor loss point (Ψ _TLP_), a well-defined physiological trait that is relatively easy to measure (Bartlett *et al.*, 2012; Petruzzellis *et al.*, 2019). Ψ _TLP_ is a good predictor of stomatal closure (Martorell *et al.*, 2015) and the onset of molecular responses to stress (McAdam and Brodribb, 2018). The latter is possibly a result of the activation of membrane proteins when turgor loss leads to membrane shrinkage. It is important to reiterate that this trait is very plastic and can be shifted by up to 1 MPa along the season or in response to water deficit (Alsina *et al.*, 2007; Martorell *et al.*, 2015). The extent of Ψ _TLP_ plasticity is likely to be a good indicator for the capacity of a cultivar to osmotically adjust to withstand low water potentials.
(iv) Root volume, one of the most basic and enigmatic physiological traits. When combined with the soil retention curve, the rooting volume could be used to determine the soil water reservoir that is available to the vine. Alsina *et al.* (2011) found that drought-adapted rootstocks tend to have deeper roots. Currently, reliable methods to estimate rooting volume are being developed, such as 3D soil tomography (Zhu *et al.*, 2014) and water isotope quantification (Savi *et al.*, 2018), which may provide insight into this trait in the future.

By considering all of these core traits together, one could more holistically evaluate a vine’s drought tolerance. Here we introduce the idea of a vine’s ‘stress distance’. The stress distance would be a quantitative value with the units of ‘time’ that would represent the amount of time that a particular vine could go without water, under a given set of environmental conditions, until it reached the critical water potential threshold, or Ψ _crit_. The stress distance can be described quantitatively as:

$$\begin{array}{l} stress\;distance=\frac{root\;volume\;\times \;\overset{\Psi_{crit}}{\underset{\Psi_{0}}{\int }}soil\;capacitance\;(\%)}{E_{max}\;\times \;\overset{\Psi_{crit}}{\underset{\Psi_{0}}{\int }}g_{s}\;(\%)} \end{array}$$

where Ψ _0_ is the initial water potential and Ψ _crit_ is evaluated from the *g*_s_–Ψ _leaf_ relationship or as Ψ _TLP_.

**Fig. 4.**
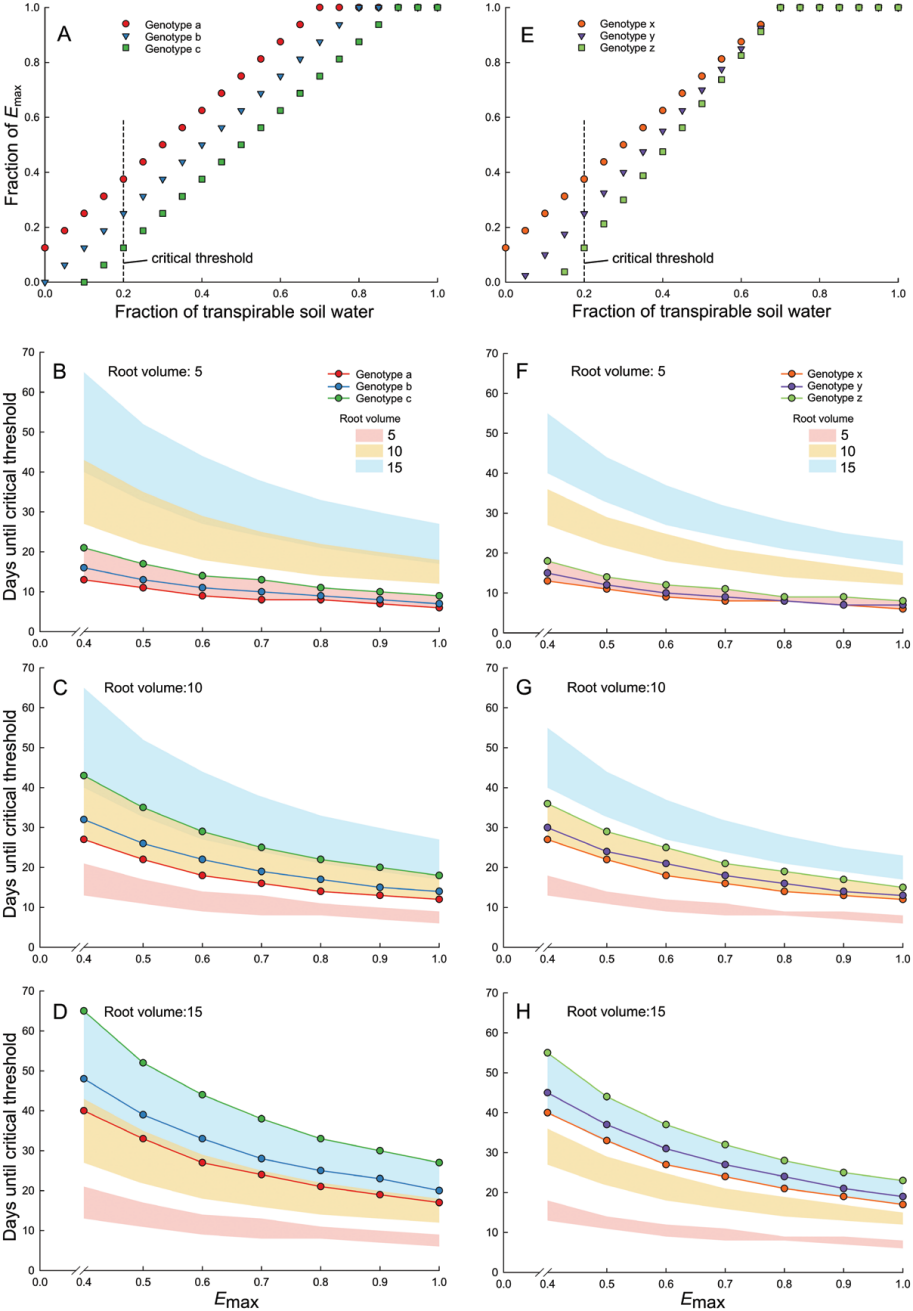
Comparing the relative importance of stomatal regulation, root volume, and maximum transpiration (*E*_max_) in determining the amount of time until a critical threshold (here defined as 20% of transpirable soil water) is reached. We created six generic genotypes with different stomatal control behaviors, three (A–D) where only the break point varies (i.e. when stomata regulation starts), and three (E–H) where only the slope varies (i.e. the speed at which stomata close). We calculated the days until the critical threshold would be reached (i.e. the stress distance) varying the root volume with generic values of 5 (B, F), 10 (C, G), 15 (D, H), and *E*_max_. The shading in the panels is provided for reference and represents the range of values across the three genotypes for a given root volume.

This is an oversimplification, but a useful one, and is not intended to supersede more rigorous attempts at modeling vine water use (Lebon *et al.*, 2003; Peccoux *et al.*, 2018; Zhu et al., 2018, 2019). Using this framework in a simplified thought experiment gives insight into the relative importance of the various traits. Figure 4 illustrates how the stress distance would change when varying stomatal regulation, root volume, and *E*_max_ under generic scenarios (Ψ _crit_ does not vary in this example). High *E*_max_ decreases the relative impacts of varying stomatal regulation and root volume. The importance of *E*_max_ is reinforced by the two examples of varied stomatal control, in which changing the point at which *E* begins to be regulated (Fig. 4A–D) has a greater impact on increasing the stress distance than changing the slope of the regulation (Fig. 4E–H). The idea of a variable *E*_max_ is nuanced and could reflect innate differences between genotypes when *E*_max_ (or *g*_max_) is considered on a per unit leaf area basis (Levin *et al.*, 2019; Dayer *et al*., 2020), and/or could reflect differences in canopy size, architecture, and/or planting density (Lebon *et al.*, 2003; van Leeuwen *et al.*, 2019). Stomatal regulation, on the other hand, becomes increasingly important as the stress distance increases, through decreased *E*_max_ and/or increased root volume (Fig. 4).

The stress distance thought experiment provides several interesting conclusions regarding what constitutes drought tolerance in grapevine. The first is to highlight the importance of *E*_max_. This reinforces traditional viticultural practices for dry climates, where low planting densities (theoretically increasing the rooting volume) and training systems favoring smaller canopies (theoretically decreasing *E*_max_) have traditionally been used. It also suggests that within a single season, reducing canopy size (i.e. pruning) could provide an excellent emergency strategy for vineyards that face extreme drought in the absence of irrigation infrastructure. The stress distance thought experiment also speaks to the impact of using genotypes with a tighter stomatal regulation, and suggests that this strategy will be much more effective if used together with strategies that slow the dry-down, by decreasing maximum water use and/or increasing water availability to the vine. Slowing the dry-down has the added advantage that it provides more time for the vine to acclimate (e.g. decrease Ψ _TLP_, Hochberg *et al.*, 2017*a*).

### Berry drought responses: hydraulics, yield, and metabolism

#### Berries hydraulics

Red winegrape production is peculiar in that growers often hope for, or purposefully apply, water deficits. Water deficits increase red winegrape quality through numerous mechanisms that include increasing the accumulation of quality-related metabolites (detailed below) and decreasing berry size leading to an increased ratio of berry skin (where many quality related metabolites accumulate, e.g. phenolics) to flesh. Berry fresh weight largely results from the accumulation of water, and to a lesser extent sugar, so how berries are hydraulically integrated with the parent vine is critical in controlling growth, ripening, and the response to water deficit.

**Fig. 5.**
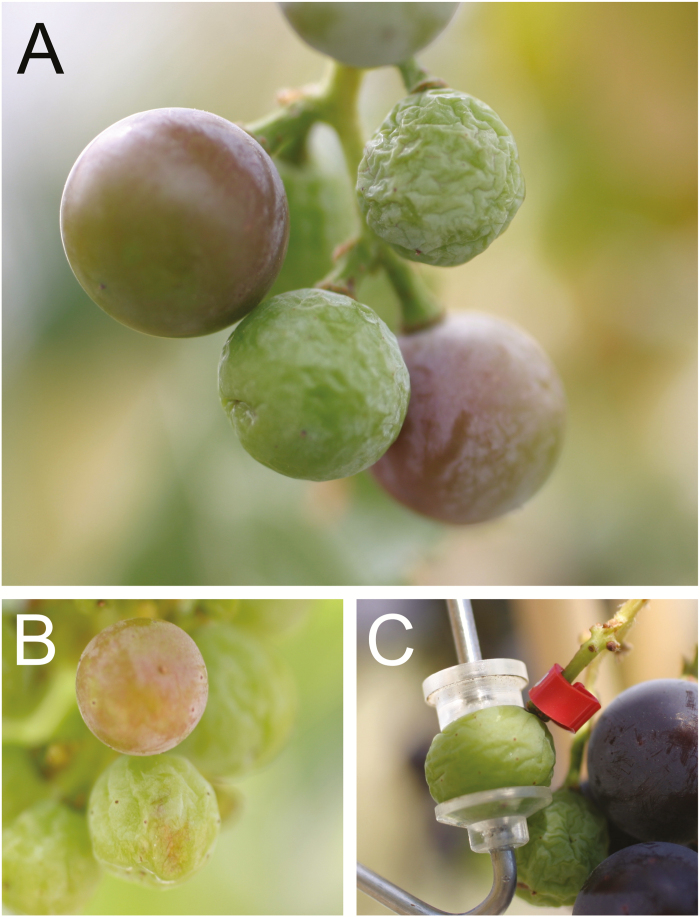
Examples of the hydraulic buffering that occurs at veraison in grapevine. When a vine is subjected to water deficit, pre-veraison berries (i.e. green berries) are sensitive to drought-induced shriveling, and berries that are either undergoing veraison (A, B, reddish-green berries) or post-veraison (C, purple berries) are insensitive. Vines shown are under a very severe water deficit (Ψ _stem_<−1.5 MPa). Photos courtesy of Markus Keller.

Berry water relations are markedly different before and after the onset of ripening (veraison). Before veraison berries are hydraulically connected to the vine, and although they have very few, or no, functional stomata they exhibit high rates of cuticular transpiration (Blanke and Leyhe, 1987; Zhang and Keller, 2015). Pre-veraison berries are also sensitive to changes in the vine’s Ψ and can experience drought-induced shriveling (Fig. 5; Hardie and Considine, 1976; Greenspan *et al.*, 1994). After veraison the hydraulic connection between berries and the vine changes; transpiration decreases due to a reduction of the cuticular conductance—likely resulting from changes in cuticular wax composition (Rogiers *et al.*, 2004; Dimopoulos *et al.*, 2020). The pathway of water transport into the berry changes from the xylem to the phloem in concert with the sharp increase in sugar transport and accumulation (Greenspan *et al.*, 1994; Greer and Rogiers, 2009). Additionally, the hydraulic conductance of the berry and pedicel decreases (Tyerman *et al.*, 2008; Choat *et al.*, 2009; Knipfer *et al.*, 2015b). In contrast with pre-veraison berries, post-veraison berries are buffered from changes in the vine’s Ψ and largely insensitive to drought-induced shriveling (Fig. 5; Hardie and Considine, 1976; Greenspan *et al.*, 1994; Keller *et al.*, 2015).

The mechanisms that result in this hydraulic buffering of the berry are still unknown. Although studies have measured a decrease in the hydraulic conductance of the berry and pedicel, the magnitude of this decrease does not appear great enough to account for the buffering (see discussions of Choat *et al.*, 2009; Knipfer *et al.*, 2015b), and other studies have clearly demonstrated that the xylem pathway remains functional after veraison (Bondada *et al.*, 2005; Keller *et al.*, 2006; Chatelet *et al.*, 2008). It is even likely that excess water imported via the phloem could be recycled into the parent plant through the xylem (Tilbrook and Tyerman, 2009; Choat *et al.*, 2009; Keller *et al.*, 2015; Zhang and Keller, 2017). One possibility is that the decreases in hydraulic conductance have been underestimated because the viscosity of the xylem sap has not been taken into account. Grape berries accumulate an incredibly high amount of sugars (among the highest of all fruit) and studies have suggested that sugars are almost equally distributed between the berry apoplast and symplast (Wada et al., 2008, 2009). When the viscosity is taken into account the decreases in hydraulic conductance could be between ~25% (at 10 °Bx) and ~100% (at 20 °Bx) greater. Another possibility is that sugar accumulation alone could alter berry water relations in a way that creates a buffered system. The exact details are difficult to imagine, especially since turgor and elasticity of post-veraison berries are very low (Thomas *et al.*, 2006; Wada et al., 2008, 2009; Castellarin *et al.*, 2016).

#### Berry size and yield

Water deficits reduce berry size and yield, and studies have shown that the decreases are linearly related to decreases in Ψ. [Bibr CIT0076][Bibr CIT0210] reported a 41% decrease in yield per MPa decrease in Ψ and a more recent work by the same group found a 66% decrease in berry weight per MPa decrease in Ψ (Williams *et al.*, 2010). These findings are similar to the 50% average decrease in berry weight per MPa decrease in Ψ found by a recent meta-analysis (Mirás-Avalos and Intrigliolo, 2017). However, Mirás-Avalos and Intrigliolo (2017) found that this relationship was variety dependent. When taken together studies suggest that, on average, a moderate decrease in Ψ of 0.2 MPa results in a 10% yield penalty. The severity, duration, and timing of the water deficit greatly impact the berry’s response in terms of size and metabolic adjustment. For example, there is evidence that the reduction of berry size and yield is greater with pre-veraison water deficits (Hardie and Considine, 1976; Matthews and Anderson, 1989).

#### Water deficit and berry metabolism

**Fig. 6.**
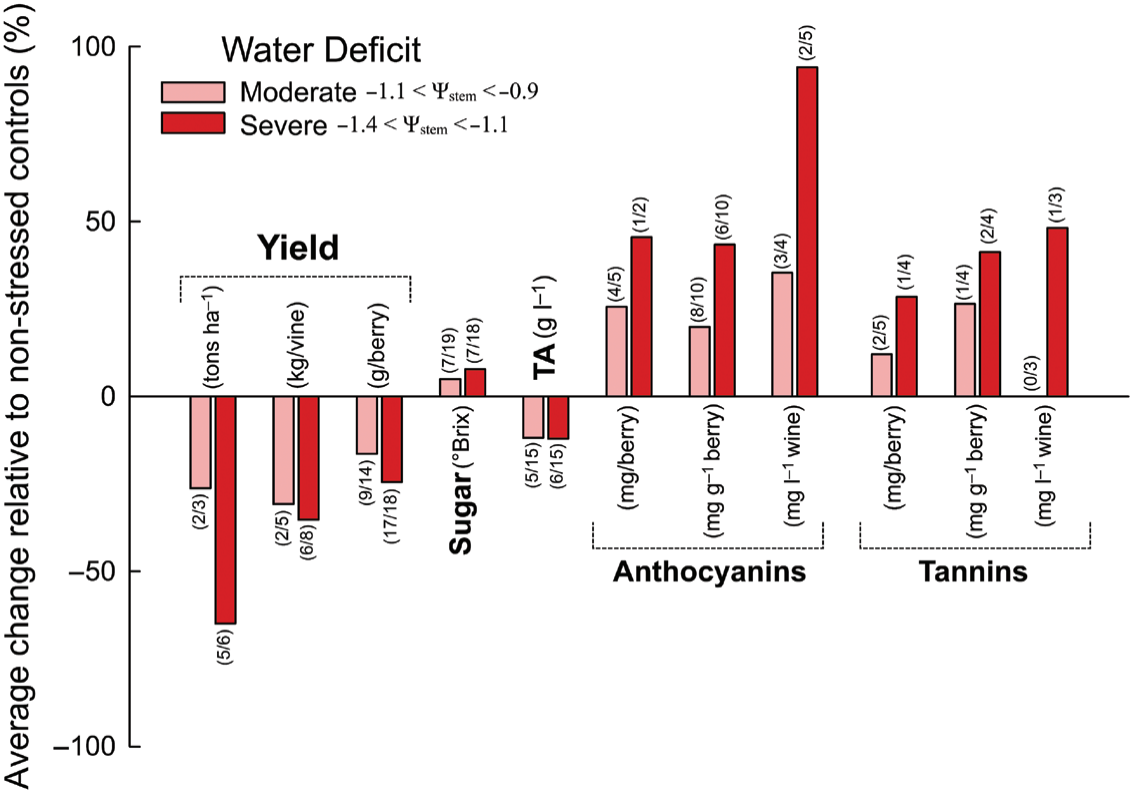
Meta-analysis of 18 published studies investigating berry (and sometimes wine) composition changes under water deficit. Experiments were divided into those applying either a moderate (pink bars) or a severe (red bars) water deficit; the corresponding Ψ _stem_ ranges (values represent mean Ψ _stem_ across the entire study period) are noted in the figure. The proportion of study years demonstrating statistically significate effects are noted above each bar in parentheses. The bars represent the average percentage change (for only those studies showing statistically significant changes) of the compound relative to non-stressed controls. Each season within a study was treated separately and all references and detailed data can be found in Supplementary Table S1.

Water deficit greatly affects berry metabolism. The recent meta-analysis of Mirás-Avalos and Intrigliolo (2017) indicates that sugars and organic acids negatively and positively correlate, respectively, with Ψ. However, inconsistent results have been reported among studies. We closely analysed 18 studies performed on four major red grape varieties (Cabernet Sauvignon, Merlot, Syrah, and Tempranillo). In these studies, water potentials for well-irrigated (i.e. control) and deficit-irrigated vines were compared and water deficit levels corresponded to either moderate (−0.9<Ψ _stem_<−1.1 MPa) or severe water deficit (−1.1<Ψ _stem_<−1.4 MPa) (Fig. 6; Supplementary Table S1 at *JXB* online). Moderate deficit significantly affected sugar concentration at harvest in only 7 out of 19 experiments (seasons were independently considered within each study). In all those cases, the deficit increased the concentration (5% on average). Similar results were obtained with severe deficit (7 out of 18 studies indicated a significant increase by 7.8% in sugar concentration under deficit). Similarly, few experiments identified an effect of water deficit on titratable acidity (5 out of 15 for moderate deficit and 6 out of 15 for severe deficit). In all cases but one, titratable acidity was reduced (approx. −12% for both moderate and severe deficit). This analysis indicates that varieties respond differently to water deficit (for instance, Merlot was more sensitive than Cabernet Sauvignon; Supplementary Table S1) and that seasonal conditions affect the responses as suggested by Herrera *et al.* (2017).

Like the osmotic adjustment observed in leaves under drought (discussed above), berries also accumulate osmolytes in response to water deficit. Nitrogen metabolism seems to play a central role in this process in which water deficit increases the concentration of the amino acids proline, leucine, isoleucine, and valine in parallel with the up-regulation of genes related to their biosynthesis (Deluc *et al.*, 2009; Savoi *et al.*, 2017; Canoura *et al.*, 2018). The accumulation of these osmolytes in response to water deficit can vary between varieties (Deluc *et al.*, 2009; Bouzas-Cid *et al.*, 2018). The role of amino acids in adjusting berry osmotic potential under drought has to be integrated with our current knowledge of the predominant molecules driving berry osmotic potential during ripening: malate, tartrate, glucose, fructose, and potassium before veraison, and mostly glucose and fructose after veraison (Wada *et al.*, 2008).

#### Flavonoids

Several specialized (also known as secondary) berry metabolites strongly respond to abiotic stressors such as water deficit. Among these metabolites, flavonoids and volatile organic compounds (discussed below) are the most important since they largely contribute to the grape and wine flavor and quality.

Water deficit results in red wines with a higher concentration of anthocyanins, more intense pigmentation, and color shifts towards bluer hues (e.g. Herrera *et al.*, 2015; Cáceres-Mella *et al.*, 2018) due to increases in the anthocyanin concentration in grapes and wines (e.g. Matthews and Anderson, 1988; Chapman *et al.*, 2004; Castellarin et al., 2007*a*, *b*; Deluc *et al.*, 2009; Koundouras *et al.*, 2009; Intrigliolo *et al.*, 2012; Savoi *et al.*, 2017). This trend was consistent among the grape varieties in our meta-analysis (Fig. 6; Supplementary Table S1). However, there are examples of studies that have shown no significant changes in anthocyanin levels under water deficit, so this response, although common, is not universal (Bonada *et al.*, 2015; Herrera *et al.*, 2017; Brillante *et al.*, 2018). From the numerous studies on the subject, it emerges that seasonal weather conditions, grapevine variety, and the magnitude and timing of the water deficit dictate the precise effects on berry and wine anthocyanins (Koundouras *et al.*, 2009; Bucchetti *et al.*, 2011; Shellie and Bowen, 2014; Pinasseau *et al.*, 2017; Buesa *et al.*, 2019). For instance, water deficit had a greater impact on anthocyanin accumulation in Syrah than in Cabernet Sauvignon berries and induced the production of acylated anthocyanins in Cabernet Sauvignon while reducing the same compounds in Syrah (Hochberg *et al.*, 2015a). Consistent with the observed changes in metabolism, several genes of the phenylpropanoid and flavonid pathway are up-regulated under water deficit (Castellarin et al., 2007*a*, *b*; Deluc *et al.*, 2009; Martínez-Lüscher *et al.*, 2014; Savoi *et al.*, 2017). Some of these genes control the branching of the pathway. The increased expression of 3′,5′-flavonoid hydroxylases and methyltransferases under water deficit resulted in a relative increase in the concentration of tri-substituted and methoxylated anthocyanins, respectively (Castellarin et al., 2007*a*, *b*; Martínez-Lüscher *et al.*, 2014; Savoi *et al.*, 2017). Higher levels of methylation increase the stability of anthocyanin (Sadilova *et al.*, 2006), and higher levels of hydroxylation shift the pigment hue to more purple-blue (Tanaka and Brugliera, 2013).

The impact of drought on berry flavan-3-ols and proanthocyanidins (also known as tannins) still remains unclear as contrasting results have been reported among studies (Castellarin *et al.*, 2007a; Deluc *et al.*, 2009; Ollé *et al.*, 2011; Hochberg *et al.*, 2015a; Casassa *et al.*, 2015; Savoi *et al.*, 2017). Pre- and post-veraison application of water deficit increased proanthocyanindin levels in Syrah and Cabernet Sauvignon berries, but only transiently, and at harvest no differences were observed (Castellarin *et al.*, 2007a; Ollé *et al.*, 2011). Other studies in Cabernet Sauvignon showed that water deficit increased proanthocyanidin content corresponding to the up-regulation of the flavan-3-ol- and proanthocyanindin-related genes *VviLAR2* and *VviMYBPA1* (Casassa *et al.*, 2015; Cáceres-Mella *et al.*, 2017). A recent study that compared the berry phenolic profile of 279 *Vitis vinifera* varieties to water deficit indicated that berry polyphenol response is not consistent among varieties, with different molecular families affected either positively or negatively between the different varieties (Pinasseau *et al.*, 2017). However, a general increase in the mean degree of polymerization and percentage tri-hydroxylation of proanthocyanindins was observed, and similar effects on the degree of polymerization were observed in other studies (Ollé *et al.*, 2011; Cáceres-Mella *et al.*, 2017; García-Esparza *et al.*, 2018). In contrast, Yu *et al.* (2016) showed that in Merlot berries there was no effect of water deficit on the skin proanthocyanidin concentration and degree of polymerization.

Flavonols are another major class of flavonoids that accumulate in grapes. Most studies have shown no effect of water deficit on flavonol concentration (Castellarin *et al.*, 2007a; Martínez-Lüscher *et al.*, 2014; Yu *et al.*, 2016; Savoi *et al.*, 2017); nevertheless, several studies have shown transient increases in the expression of the flavonol synthase (*VviFLS*) genes under water deficit (Ojeda *et al.*, 2002; Castellarin *et al.*, 2007*b*; Savoi *et al.*, 2016), and increases in flavonol concentration during berry ripening have also been reported (Ojeda *et al.*, 2002; Deluc *et al.*, 2009; Savoi *et al.*, 2016). In addition to having direct effects on berry metabolism, water deficit reduces canopy growth, potentially increasing the exposure of clusters to sunlight (Castellarin *et al.*, 2007*b*). Flavonol biosynthesis is particularly responsive to light and UV exposure (reviewed in Teixeira *et al.*, 2013), so inconsistencies among studies might be related to differences in the impact of the water deficit on canopy structure. Moreover, Deluc *et al.* (2009) suggested that the increase in flavonol production observed in white varieties (e.g. Chardonnay) exposed to water stress might be an alternative strategy for photo-protection as white berries lack anthocyanins.

Inconsistencies among studies have also been found when analysing the impact of water deficit on stilbene accumulation, indicating a potential variety-specific response. Deluc *et al.* (2011) showed that water deficit increased the accumulation of *trans*-piceid, a glucosylated form of resveratrol, in Cabernet Sauvignon but not Chardonnay. Other studies have also reported variety-specific effects of water deficit on increases in stilbene concentration (Versari *et al.*, 2001; Vezzulli *et al.*, 2007). However, decreases in stilbene biosynthesis under water deficit have also been demonstrated, perhaps resulting from the competition for precursors between the stilbenoid and flavonoid pathway (Hochberg *et al.*, 2015a; Savoi *et al.*, 2017).

#### Volatile organic compounds

Wine aroma is affected by the vine water status (Chapman *et al.*, 2004; Bonada *et al.*, 2015; Herrera *et al.*, 2015; Picard *et al.*, 2017), but despite the relevance of the topic for commercial production, the impact of water deficit on the accumulation of wine aroma precursors has rarely been investigated. In red grapes, water deficit results in wines with a higher concentration of volatiles (Talaverano *et al.*, 2017) and distinctive organoleptic features such as a higher level of red/black berry, jam/cooked berry, dried fruit, and raison aromas (Chapman *et al.*, 2004; Bonada *et al.*, 2015; Herrera *et al.*, 2015). Riesling wines produced under a moderate water deficit had more pronounced citrus, apple, and pear aromas (Marciniak *et al.*, 2013).

Terpenoids are a major class of secondary metabolites that include several key aromatics for grapes and wines, such as monoterpenes, sesquiterpenes, and C_13_-norisoprenoids, which contribute to floral and fruity aromas. For example, monoterpenes are responsible for the typical aromas of Muscat, Riesling, Viognier, and Gewürztraminer (Robinson *et al.*, 2014; Siebert *et al.*, 2018). Water deficit increased the concentration of terpene alcohols in both white and red grapes (Song *et al.*, 2012; Savoi *et al.*, 2016), partially through the up-regulation of key methylerythritol phosphate pathway (MEP) genes including terpene synthases (Deluc *et al.*, 2009; Savoi *et al.*, 2016). Interestingly, many of these responsive genes were enriched for drought-related regulatory elements in their promoter regions (Savoi *et al.*, 2016). However, the terpene response to water deficit might not be common for all varieties as recent studies also report decreases in terpene concentration under water deficit (Brillante *et al.*, 2018; Bouzas-Cid *et al.*, 2018). Carotenoids and their volatile C_13_-norisoprenoid degradation products were also increased by water deficit (Bindon *et al.*, 2007; Song *et al.*, 2012). The increase of C_13_-norisoprenoids may be due to a larger production of the carotenoid precursors (Bindon *et al.*, 2007) and/or a larger breakdown of carotenoids, as the gene encoding a key norisoprenoid synthesis enzyme, cleavage dioxygenase, was up-regulated by water deficit (Savoi *et al.*, 2016).

Methoxy-pyrazines are compounds that impart a herbaceous or ‘green’ aroma to grapes and wines. Research has found that water deficit can reduce the levels of 3-isobutyl-2-methoxy-pyrazine in Syrah wine (Brillante *et al.*, 2018). Despite the lack of information on whether and how water deficit affects methoxy-pyrazine production and/or degradation, we hypothesize that water deficit indirectly reduces methoxy-pyrazine by limiting canopy growth, increasing cluster exposure, and thus increasing light intensity and temperature (Ryona *et al.*, 2008; Šuklje *et al.*, 2012; Mosetti *et al.*, 2016; Gregan and Jordan, 2016). Water deficit also decreases other compounds imparting herbaceous aromas, such as the C_6_ compounds hexanal, trans-2-hexenal, and 1-hexanol (Song *et al.*, 2012; García-Esparza *et al.*, 2018). However, an increase of such compounds under water deficit or specific regulated deficit irrigation strategies was also shown (Talaverano *et al.*, 2018; Vilanova *et al.*, 2019). Other grape volatile aldehydes and alcohols that impart both green and fruity aromas, such as 2-heptenal, 1-octen-3-ol, 1-octen-3-one, 2-octenal, nonenal, and nonanol, were induced under water deficit (Savoi et al., 2016, 2017).

#### Interaction with other factors: environment and phenology

Disagreement among studies indicates that varieties often display different berry metabolic responses to the same abiotic stresses (Deluc et al., 2009, 2011; Hochberg *et al.*, 2015a; Savoi *et al.*, 2017). These inconsistencies could be due to multiple factors. Studies have shown that the timing of water deficit differentially impacts grape and wine quality (Matthews and Anderson, 1988; Castellarin *et al.*, 2007a). Early water deficits prior to veraison have a larger effect on berry size and thus a stronger potential for increasing the concentration of the phenolic compounds (Matthews and Anderson, 1988; Wong *et al.*, 2016). Interestingly, studies also indicate that water deficit continues to effect the biosynthesis of anthocyanins even after the deficit was relieved (Castellarin *et al.*, 2007a), suggesting that the signaling pathways and hormones involved in the berry response remain activated beyond the duration of the stress.

Variety (Deluc *et al.*, 2009; Hochberg *et al.*, 2015a), rootstock (Berdeja *et al.*, 2015), soil structure (Tramontini *et al.*, 2014), and other environmental variables such as light and temperature (Herrera *et al.*, 2017) also play important roles in modulating the composition and quality of grapes grown under water deficit (Zarrouk *et al.*, 2016). The complex interaction between factors is well illustrated by the example of grape berry phenolics. Water deficit enhances berry color, but high berry temperature induces the degradation of anthocyanins, limiting and sometimes even reversing the positive effects of water deficit (Bonada *et al.*, 2015; Zarrouk *et al.*, 2016; Cooley *et al.*, 2017; Herrera *et al.*, 2017). These interactions extend to wine sensory traits, including modulating tannin structure as well as floral and fruity aromas (Bonada *et al.*, 2015).

In order to improve our knowledge of berry metabolic response to water deficit, and the underlying mechanisms in relation to variety and environment, controlled environmental studies should be performed in which varieties are compared in identical conditions with appropriate experimental designs. Field studies, particularly in commercial vineyards, provide a wealth of knowledge, but within this setting, it is difficult to compare multiple genotypes, or characterize the interaction of irrigation regimes with other environmental variables.

#### Water deficit signaling in berries

Research suggests that both ABA-dependent and ABA-independent mechanisms are involved in the regulatory network controlling changes in berry metabolism under water deficit. ABA-dependent pathways are evident considering the role of ABA as a cornerstone in controlling the onset of ripening in non-climacteric fruits such as grape (Wheeler *et al.*, 2009; Lacampagne *et al.*, 2010; Giribaldi *et al.*, 2010; Gambetta *et al.*, 2010) and its role in regulating phenolic biosynthesis even under non-stressed conditions (e.g. Gagné *et al.*, 2011; Villalobos-González *et al.*, 2016). In berries, water deficit increases the expression of ABA biosynthetic genes (Deluc *et al.*, 2009; Savoi *et al.*, 2017) and ABA concentration (Deluc *et al.*, 2009; Niculcea *et al.*, 2014). The promoters of many water deficit-induced genes are enriched for ABA-related *cis*-regulatory elements (Savoi *et al.*, 2016) including those for biosynthesis of aroma compounds such as terpenes (discussed above). In addition to ABA-dependent pathways, studies also suggest that ABA-independent pathways act in response to water deficit in the grape berry. Notably, many components of the ethylene signaling pathway are strongly up-regulated by a water deficit during ripening (Savoi *et al.*, 2017), suggesting ethylene may play an important role in both climacteric and non-climacteric fruit ripening (Sun *et al.*, 2010; Chen *et al.*, 2018). It remains to be determined how these molecular signals, which could be endogenous (arising through *de novo* synthesis in berries) or exogenous (arriving from the parent plant), are integrated and how the hydraulic connection of the berry with the parent plant affects this integration.

#### Integrating berries with the vine: relationships between source–sink and water

In the previous sections we have discussed the effects of water deficit on grape berry growth and metabolism, which in turn should impact the source–sink relations of the plant. The relationships between source–sink and plant water use are poorly studied, but it has been suggested that fruit load can impact gas exchange of the parent plant (Körner, 2015), specifically that decreasing fruit load (i.e. increasing source to sink ratio) would result in a decrease in *g*_s_ and assimilation. Under water deficit, reductions in growth are greater than reductions in assimilation, which leads to the accumulation of sugars in leaves (Muller *et al.*, 2011). This increased sugar accumulation in leaves could ultimately down-regulate *g*_s_ and leaf hydraulic conductance (Kelly et al., 2013, 2017). This hypothesis is supported from many studies that found that fruit removal led to lower gas exchange in plum (Gucci *et al.*, 1991), olive (Bustan *et al.*, 2016), avocado (Silber *et al.*, 2013), and coffee (Franck *et al.*, 2006). In many other species, sink limitation through girdling restricted gas exchange, providing additional support for the hypothesis. In grapevines, Williams *et al.* (2000) showed that girdling reduced *g*_s_ from 500 to 300 mmol m^−2^ s^−1^ and increased Ψ _leaf_ by 0.3 MPa. Similarly, cluster thinning decreased assimilation (Iacono *et al.*, 1995), and in the opposite sense, decreasing source to sink ratio by defoliating two-thirds of the leaves increased stomatal conductance by 20% (Hunter and Visser, 1989). Other studies in grape have found no effect of fruit load on vine gas exchange. Fruit removal at different stages of development in plants growing in lysimeters did not affect whole plant transpiration (Y. Netzer *et al*., personal communication). Additionally, several studies that compared gas exchange before and after harvest did not find major differences in *g*_s_ (e.g. Munitz *et al.*, 2017). This limited number of studies highlight our inability to resolve berry effects on grapevine water demand and the need for more studies that combine carbon allocation with hydraulics.

## Conclusions

Our knowledge on grapevine drought stress physiology has increased tremendously in recent years. However, we still lack a mechanistic understanding of many drought responses, which prevents accurate modeling under future climatic scenarios. The remaining questions are numerous. For example, what are the genetic determinants of key drought tolerance traits such as *E*_max_, *g*_s_ regulation, Ψ _TLP_ acclimation, rooting behavior, and the plasticity of xylem structure that directly impact productivity and vulnerability? How plastic are these traits within a single genotype? To what extent are key drought tolerance traits interconnected genetically and to what extent can they be disentangled for breeding purposes? Can scion–rootstock interactions be better exploited to modulate drought tolerance? Future research needs to aim to answer these questions and should consider interactions with other stressors (e.g. heatwaves, radiation, biotic stressors) that are likely to co-occur in the field.

Climate change is threatening our eco- and agrosystems and we still lack the knowledge to accurately predict how crops will respond to these new challenges. For viticulture, the threat of climate change produces anxiety for individual growers and winemakers, and regional and national governments, all of which rely on this economically and culturally important crop. A clear understanding of grapevine responses to water deficit is critical in addressing these concerns, especially in increasing the efficiency and resiliency of viticultural practices and guiding the development of drought-tolerant varieties and rootstocks.

## Supplementary data

Supplementary data are available at *JXB* online.

Table S1. Data and references of the 18 published studies used in a meta-analysis investigating berry and wine composition changes under water deficit.

eraa245_suppl_Supplementary_Table_S1
