# Corrigendum to: The physiology of drought stress in grapevine: towards an integrative definition of drought tolerance

**DOI:** 10.1093/jxb/eraa313

**Published:** 2020-07-27

**Authors:** Gregory A Gambetta, Jose Carlos Herrera, Silvina Dayer, Quishuo Feng, Uri Hochberg, Simone D Castellarin

**Affiliations:** 1 EGFV, Bordeaux-Sciences Agro, INRA, Université de Bordeaux, ISVV, chemin de Leysotte, Villenave d’Ornon, France; 2 Institute of Viticulture and Pomology, Department of Crop Sciences, University of Natural Resources and Life Sciences Vienna (BOKU), Tulln, Austria; 3 Wine Research Centre, Faculty of Land and Food Systems, The University of British Columbia, Vancouver, BC, Canada; 4 ARO Volcani Center, Institute of Soil, Water and Environmental Sciences, Rishon Lezion, Israel

*Journal of Experimental Botany*, Advance Access publication: 20 May 2020, doi: 10.1093/jxb/eraa245

An equation in the above article has been corrected as below.

Incorrect equation:

$$\begin{array}{l} \;\;\;Stress\;distance=\frac{E_{max}\times (\frac{E_{{\;\Psi \;}_{0}}}{E_{max}})}{Root\;volume\times \overset{{\;\Psi \;}_{crit}}{\underset{{\;\Psi \;}_{0}}{\int }}Soil\;capacitance} \end{array}$$

Corrected equation:

$$\begin{array}{l} stress\;distance=\frac{root\;volume\;\times \;\overset{\Psi_{crit}}{\underset{\Psi_{0}}{\int }}soil\;capacitance\;(\%)}{E_{max}\;\times \;\overset{\Psi_{crit}}{\underset{\Psi_{0}}{\int }}g_{s}\;(\%)} \end{array}$$

